# Tracheotomy

**Published:** 1845-06

**Authors:** 


*Tracheotomy—Removal of a pipe stem* $1\frac{3}{4}$ *inch long—Cure.*—Dr. Hall, of Burlington, Vt., relates the following case in the last (April) number of the American Journal of the Medical Sciences. The subject was a little boy aged four years, who drew the pipe stem into his windpipe while holding and drawing air through it between his lips. Every thing being in readiness the operation was thus executed:

"I made the incision through the skin and adipose substance from the os hyoides downwards to just below the thyroid cartilage, the lad all the while screaming, "Have you got the pipe-stem?" In making this incision I divided a vein of considerable size at the lower angle of the wound, which bled rather freely for some minutes. This was taken up, and a ligature applied by my assistants. The bleeding being arrested, and the trachea laid bare, I made a slight incision through its upper ring, when the air and spray burst from the interior. The child was now turned upon his left side, to prevent the oozing of blood into the trachea, and the perforation was immediately slit down with a probe-pointed bistoury, the length of the external incision being about $2\frac{1}{2}$ inches. The integuments were now drawn apart with broad blunt hooks by Dr. Hatch, and search was made by Dr. Marsh for the foreign body with a probe, it not being thrown out, as is sometimes the case with lighter substances, by the forcible exit of air and phlegm in coughing. After some minutes search the pipe-stem was drawn out upon the point of the probe, it having sunk by its gravity into the right bronchus, where it was detected and extracted by the probe which had passed into its hollow. While this was being done, respiration was performed wholly through the artificial orifice, and, of course, the voice was completely hushed. The face and countenance exhibited a deep flush, with a sort of painful scowl, the eyes partly closed, and the features somewhat distorted; but the boy was now perfectly quiescent. Having removed the foreign body, the lips of the incision were drawn together by sutures and sticking plaster; but the mucous membrane of the bronchia, trachea and larynx had become somewhat thickened by the irritation, and for thirty-six hours afterwards a part of the breath was forced out between the stitches at each expiration. This could not be controlled or impeded without the most imminent danger of fatal suffocation. A dose of calomel was administered that evening, and the patient was well nursed by his friends through the night. Next morning a cathartic operation had been procured, and it was evident that the deposition of lymph in the part was arrested, for the breathing was less laborious, and all the alarming symptoms were relieved. A powder of nitre, ipecac., and a litle opium was now directed to be given once in six hours, to allay the irritation and act as an expectorant. After two days this was discontinued, respiration having in a great measure become re-established. The child's recovery was speedy and complete.

